# Liver Surgery for Hepatocellular Carcinoma: Laparoscopic versus Open Approach

**DOI:** 10.4061/2011/596792

**Published:** 2011-05-16

**Authors:** C. G. Ker, J. S. Chen, K. K. Kuo, S. C. Chuang, S. J. Wang, W. C. Chang, K. T. Lee, H. Y. Chen, C. C. Juan

**Affiliations:** ^1^Division of HBP Surgery, Chung-Ho Memorial Hospital, Institute of Medicine, Kaohsiung Medical University, Kaohsiung 80756, Taiwan; ^2^Department of Surgery, Yuan's General Hospital, No. 162, Cheng-Kong 1st Road, Kaohsiung 80249, Taiwan

## Abstract

In this study, we try to compare the benefit of laparoscopic versus open operative procedures. *Patients and Methods*. One hundred and sixteen patients underwent laparoscopic liver resection (LR) and another 208 patients went for open liver resection (OR) for hepatocellular carcinoma (HCC). Patients' selection for open or laparoscopic approach was not randomized. *Results*. The CLIP score for LR and OR was 0.59 ± 0.75 and 0.86 ± 1.04, respectively, (*P* = .016). The operation time was 156.3 ± 308.2 and 190.9 ± 79.2 min for LR and OR groups, respectively. The necessity for blood transfusion was found in 8 patients (6.9%) and 106 patients (50.9%) for LR and OR groups. Patients resumed full diet on the 2nd and 3rd postoperative day, and the average length of hospital stay was 6 days and 12 days for LR and OR groups. The complication rate and mortality rate were 0% and 6.0%, 2.9% and 30.2% for LR and OR groups, respectively. The 1-yr, 3-yr, and 5-yr survival rate was 87.0%, 70.4%, 62.2% and 83.2%, 76.0%, 71.8% for LR and OR group, respectively, of non-significant difference. From these results, HCC patients accepted laparoscopic or open approach were of no significant differences between their survival rates.

## 1. Introduction

Hepatocellular carcinoma (HCC) is a well-known disease in Taiwan. To date, the literature on laparoscopic hepatic surgery is not common and believed this technique is an innovation [1, 2]. In 1998, we started to apply laparoscopic approach for liver surgery on liver cancer [2]. In the study of Santambrogio et al. [3], evaluation by laparoscopic echography is indispensable to guarantee precise determination of the segmental tumor location and the relationship of the tumor to adjacent vascular and biliary structure which were important in the perioperative liver dissection.

With the improvement of laparoscopic technique and the development of new technology and equipment, laparoscopic liver resection is feasible and safe in experienced surgeons. In 2000, Descottes et al. [4] had reported right liver lobectomy and believed the use of this new technical approach offers many advantages but require extensive experience in hepatobiliary surgery and laparoscopic skills. In addition, the caudate lobe alone could be removed without scarifying other parts of the liver reported by Dulucq et al. [5]. Therefore, the laparoscopic technique was accepted for major liver resection gradually in some institutions [6]. 

Unlike laparoscopic cholecystectomy, laparoscopic hepatectomy has technical difficulties. The expansion of laparoscopic liver surgery will depend on the ability of expert surgeons and technological advances to address the management of bleeding and hemostasis [7]. As we had known, the open hepatic resection by large skin incision causes severe postoperative pain and longer recovery time usually. In addition to the benefits shared by all laparoscopic procedures, laparoscopic liver surgery also has theoretical advantages in some patients of HCC. Therefore, the aim of this study is to compare the results of laparoscopic procedure with open technique in the patients of HCC.

## 2. Patients and Methods

### 2.1. Patients' Data and Indications

One hundred and sixteen patients (92 male and 24 female) were encountered and underwent laparoscopic liver resection and another 208 patients (156 male and 52 female) went for traditional resection un-randomized from 1998 to 2006. The criteria for liver resection were HCC with final pathological diagnosis. The basic data and underlying condition of liver diseases were shown in Table 1.

### 2.2. Laparoscopic Approach Procedures

 Patients were in supine position under general anesthesia and the trocar insertion sites depended on the site of tumor. Usually, it was necessary to insert four trocars to have an optional operative manipulation. The first trocar was placed by small incision below the umbilicus technique for pneumo-peritoneum creation. The abdominal pressure was maintained low at the level of 8–12 mmHg in addition to abdominal lifting if necessary. The general condition of the liver could be evaluated directly from the laparoscopic examination and then to decide the following procedure. The site or extension of the tumors and its relationship to the vasculature were confirmed by laparoscopic ultrasonography. The line of intended transection and tumor feeding vessels and hepatic veins were marked on the liver surface with diathermy. Microwave coagulation along the resection line was performed first before dissecting the liver parenchyma. With this technique, risk of bleeding will be less during dissection. For the left-sided resections, the round, Falciform, and left triangular ligaments and the lesser omentum were divided. All the transection lines were punctured with laparoscopic microwave tissue coagulator to minimize bleeding during the liver dissection. Ultrasonic dissector system (CUSA) was used and branched vessels, and ducts were clipped and transected. The critical point at the left hepatic artery required double clipping. However, the left portal vein and left hepatic vein were ligated with silk and large clips. The surgical field was irrigated and checked bleeders or bile leak, and residual fluid was removed by suction. The electric coagulator was applied for ensuring hemostasis on the resection surface. After dissecting the left liver completely, the specimen could be removed by widening the epigastric port wound. Finally, a drainage tube was placed for postoperative drainage. The surgical procedure, postoperative course, and outpatient followup at 1, 3, and 5 years were evaluated periodically. The following data were collected prospectively: including duration of surgery, blood loss, perioperative transfusions, surgical events, postoperative complications, hospital stay, and survival rate.

### 2.3. Biostatistics Analysis

The clinical patients' features and postoperative results, all values, were expressed as means with standard deviations. The Student *t*-test and one-way analysis of variance (ANOVA) were used. The Kaplan-Meier method was employed to measure survival curve, and log-rank test was used to delineate a comparison between the survival rates of LR and OR groups. SPSS (versionb12.0) for Windows XP was used for data analysis. A *P* value of less than.05 was considered statistical significantly.

## 3. Results

### 3.1. Intraoperative Results

The laparoscopic procedure was completed in 116 patients. All patients who underwent laparoscopic liver resection included one segment or less in 97 patients and left lateral segmentectomy (removal of segment 2 & 3) in seven, left lobectomy (removal of segment 2, 3 & 4) in four, and right anterior sectorectomy in eight patients. The lesions were located in the right liver in 61 patients and in the left liver in 55 patients. The type of operations of LR and OR was shown in Table 2. Conversion to open laparotomy occurred in 6 patients (5.2%) due to the anatomic limitation. Mean tumor size measured on the surgical specimen was 2.5 ± 1.2 and 5.4 ± 3.5 cm for the LR and OR groups, respectively. A margin of at least 1 cm beyond tumor limits was obtained in our patients who underwent surgery for malignancy except the situation of the base of the tumor adjacent to the main vessels. Mean surgical time and blood loss for LR and OR is shown in Table 2. There were 8 in 116 patients (6.9%) who needed blood transfusion. There were no signs suggestive of gas embolism in any of our patients. 

### 3.2. Postoperative Results

**Figure 1:**
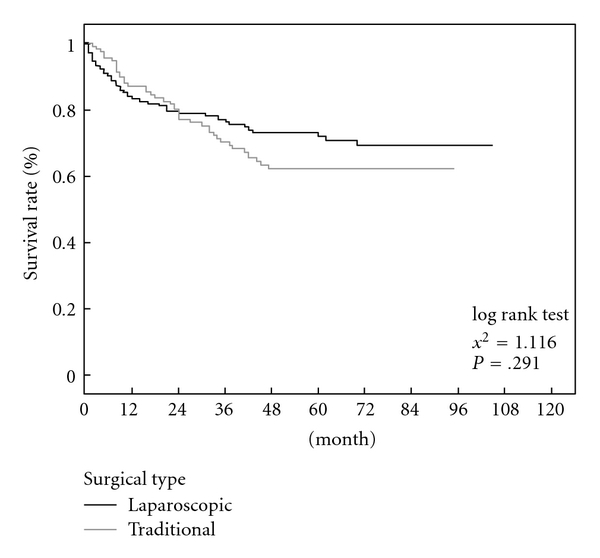
The survival curve of patients with HCC was treated by laparoscopic or open liver resection. Open method had the better result after 24 months postoperatively, but there was no significant difference totally (*P* = .291).

There was no operative mortality in LR group but 2.9% (6/202) in OR group (*P* = .092). Postoperative complications consisted of 7 and 63 patients in the LR and OR group (*P* = .001). Cirrhotic patients developed transient ascites in 2 in LR and 26 in OR group (*P* = .002) but were well controlled with medication. There were no cases of postoperative bleeding or bile leak in LR group but six and four patients in OR group. Mean hospital stay of the whole series was 6.2 ± 3 days for LR group and 12.4 ± 6.8 days for OR group with a significant difference (*P* = .001). After a mean followup of 94 months, no port-site metastasis was observed in any patient who underwent surgery for malignant disease. The 1-year, 3-year, and 5-year survival rate were found to be 87.0%, 70.4%, 62.2% and 83.2%, 76.0%, 71.8% for the LR and TR groups, respectively, of no significant difference (*P* = .291) as shown in the Figure 1. In addition, no tumor recurrence could be attributed to the laparoscopic approach during the follow-up period.

## 4. Discussion

In 1993, Nord and Brady [8] started to use laparoscope for liver surgery with the improvement of laparoscopic techniques and the development of new and dedicated technologies. Usually, limited liver resections were performed in the early stage, and advancing laparoscopic anatomical liver resections were still in development. Hilscher et al. [9] had reported their initiated formal laparoscopic liver resections in selected 20 patients and one bi-segmentectomy with unevenly results in 1998. However, most of their patients were metastatic liver tumors from the colon cancer and those livers were less cirrhosis. Far from being a routine technique in liver surgery, the laparoscopic approach to formal liver resections may be a promising procedure in selected cases where the tumor can be removed by a limited resection. Most liver surgeons are thinking about the intraoperative bleeding, and it is difficult to handle. Kaneko et al. [10] reported that three patients underwent left lateral segmentectomy and eight underwent partial hepatectomy. They still believed that the differences were seen in blood loss, and postoperative pain was minimal compared with open hepatectomy. With this technique, postoperative recovery was swift and smooth and the patients were satisfied with the operation [11]. Therefore, laparoscopic approach to left lateral sectorectomy or right hepatectomy was believed to be safe and could be considered as a routine in selected patients recently [6, 12]. Even laparoscopic redo surgery for recurrent HCC in cirrhotic patients is a feasible procedure with good short-term outcomes [13].

The most important factors in the selection of candidates for laparoscopic resection were tumor's nature (benign of malignant) and anatomical location of the tumor [14, 15]. In our experience, we believed that lesions of the left liver lobe (II and III) and the anterior sector (IVa, V, and VI) constitute a good indication for laparoscopic approach, whereas lesions of the posterior and superior liver segments (I, IVc, VII, and VIII) are technically demanding and should only be approached with extreme caution or with hand-assisted method. Another factor in the selection for laparoscopic surgery is small tumor size, as in the most of the reported series (less than 5 cm on average). They were 2.5 ± 1.2 cm in our series and most of our cases were peripheral and protruding from the hepatic parenchyma. For the traditional hepatectomy, the size of the tumor was 5.4 ± 3.5 cm (*P* < .001). Therefore, limited resection (less one segment) was found in 97 cases (83.6%) in our series, compared with that of traditional method which was 38 cases (18.3%) (*P* < .001). The mean postoperative hospital stay was 6 days and 12 days for the laparoscopic and traditional liver resection, respectively, in our series. In comparing with the report of Morino et al. [15], the postoperative hospital stay was 6.4 days (range 2–16) in the laparoscopic group, 5.7 days for noncirrhotic patients and 12.6 days for cirrhotic ones. In general, the hospital stay was short in patients treated by laparoscopic approach. Concerning the mean operating time it was 160.5 minutes and the conversion rate was 8% as reported by the National Registry reported from Spain [16]. Analgesia was administered for less than 48 hours in 55% and there was no mortality in our series. We strongly believed that the laparoscopic approach can reduce blood loss and postoperative hospital stay as well. One of the reason for this result was the limited resections were major in LR group.

Intraoperative bleeding was the most concern in this laparoscopic liver resection. In our series, eight patients (8/108) need blood transfusion. Management of bleeding during dissection requires technical experiences and more importantly, adequate preoperative evaluation is the best guarantee. The microwave coagulator and CUSA were proved useful during laparoscopic resection because it can coagulate and dissect the hepatic parenchyma to achieve adequate hemostasis during the procedures. In addition, the potential risk of gas embolism led some authors to use gasless suspension laparoscopy [17]. However, precautions such as low abdominal pressure monitoring at the level of 6–8 m are warranted [16, 18, 19]. In our experience, it will be safe if the pneumoperitoneum was set at the level of 6–10 mmHg. In addition, no port-site metastases were observed in our patients and also mentioned by Cherqui et al. [20]. 

Laparoscopic liver resection for patients of HCC with chronic liver disease is associated with lower morbidity than open resections which were usually reported [15, 21, 22], and results were similar in our series. In the report of Buell et al. [22], the complications included reoperation for hemorrhage, bile leakage, and even death from hepatic failure. Mean length of stay was 2.9 days (range = 1–14 days). In a larger series of 243 hepatectomies carried out, 113 (46.5%) were performed by laparoscopy [23]. Concerning the survival rate, another retrospective study was performed in eleven surgical centers in Europe regarding their experience with laparoscopic resection of liver malignancies, 37 patients with HCC were included, conducted by multicenter European study [24]. During a mean followup of 14 months, the 2-year disease-free survival was 44% for patients with HCC. No port-site metastases were observed during followup. The 3-year overall and disease-free survival rates for patients with HCC (mean follow-up 40 months) were 85% and 68% reported by Vibert et al. [23] and 93% and 64%, respectively, by Cherui et al. [25]. The 5-year overall cumulative survival rate for the 69 patients was 63.9%. The 5-year cumulative survival rate for patients with HCC less than 2 cm in diameter was 76.0%, and 56.3% for patients with HCC more than 2 cm in diameter [25]. It seemed to us that laparoscopic procedures were best suited for the patients of well-differentiated HCC [25, 26]. After a mean followup of 94 months in our series, there was no difference in survival rate between the two groups. The 5-year survival rate was found to be 62.2% and 71.8% for the laparoscopic and traditional methods, respectively, without significant difference (*P* = .291) in this series. It did not mean the laparoscopic method was better than that of formal open method because the tumor size was smaller in the laparoscopic group. However, the unexpected diagnosis of early HCC could be obtained only by laparoscopic technique in our experiences.

The surgical technique is an important factor in preventing intraoperative and postoperative complications in liver surgery. Laparoscopic approach in the extended hepatectomy could be performed due to the accumulation of experience and improvement of instruments nowadays [27]. Various techniques have been developed for safe dissection of the liver parenchyma. Therefore, hand-assisted laparoscopic liver resection is a more feasible procedure for removal of two segments of liver more or less [28]. Hand-port procedure could provide direct feeling with the surgeon's hand and makes possible a procedure that is almost identical to open surgery. In this method, there is a better visualization of the surgical field and dissection margin, and immediate hemostasis is also achieved by manually depressing the bleeding point. Laparoscopic liver resection using the Hand-port system is feasible for selected patients with lesions even in the posterior portion of the right hepatic lobe requiring limited resection [29]. In addition to the hand-assisted, laparoscopic assisted could be accepted recently and become more popular [30]. From the report of Inagaki et al. [31] with liver resection using the laparoscopy-assisted and total laparoscopic methods, there were no differences in the operation times, the transfusion amounts, the starting days of the patients' diets, the complication rates, or the durations of the hospital stay between the laparoscopic or open methods groups. Both the laparoscopy-assisted method and the total laparoscopic method are feasible to use for performing anatomical liver resection at present. There was no difference in the postoperative adverse event and extent of oncologic clearance due to either the improvement of surgeons' skills or the development of technology [10, 32, 33]. 

In conclusion, laparoscopic hepatectomy is beneficial for patient life quality as a minimally invasive procedure. Evolution of laparoscopic hepatectomy will depend on the development of new instrumentations. Laparoscopic hepatectomy is more feasible and with a low morbidity and mortality rate comparable to open procedures. However, prospective randomized trials are still needed to confirm those results, especially for resection of primary or metastasis liver malignant tumors.

## Figures and Tables

**Table 1:** Preoperative clinical demographic data.

Variable		Laparoscopy (*N* = 116)	Traditional (*N* = 208)	*P*
Sex	Male	92	156	.459
	Female	24	52	
Age	Total	58.31 ± 12.7	57.9 ± 11.2	.800
	Male	57.0 ± 12.2	56.9 ± 11.8	.965
	Female	63.2 ± 13.8	60.9 ± 8.6	.389
Body mass index (kg/m^2^)		25.0 ± 3.4	23.7 ± 3.4	.001*
HBsAG	No	42	84	.535
	Yes	74	124	
Anti-HCV	No	75	130	.791
	Yes	41	78	
Alpha-fetoprotein (ng/mL)		890.8 ± 3660.0	14561.3 ± 123371.4	.234
GOT (U/L)		67.8 ± 49.5	64.4 ± 52.3	.570
GPT (U/L)		64.5 ± 62.4	62.2 ± 55.0	.736
Alkaline phosphatase (U/L)		101.7 ± 58.7	123.4 ± 111.7	.052
Total bilirubin (mg/dL)		1.27 ± 1.18	0.95 ± 0.77	.003*
Albumin (gm/dL)		3.59 ± 0.61	3.86 ± 0.58	<.001*
Platelet (10^3^ uL)		41.0 ± 30.4	29.4 ± 23.1	<.001*
BUN (mg/dL)		18.2 ± 9.9	17.6 ± 10.6	.635
Serum creatinine (mg/dL)		1.14 ± 0.49	1.22 ± 1.04	.431
Prothrombin activity (%)		0.916 ± 0.090	0.952 ± 0.099	.001*
ASA class	1	51	88	.845
	2	51	100	
	3	13	19	
	4	1	1	
Child-Pugh classification	A	98	197	.008*
	B	17	10	
	C	1	1	
CLIP score		0.59 ± 0.75	0.86 ± 1.04	.016*
TNM stage	I	53	84	.001*
	II	58	76	
	III	32	38	
	IV	2	10	

**Table 2:** Comparative data of laparoscopy and traditional groups.

Variable		Laparoscopy (*N* = 116)	Traditional (*N* = 208)	*P*
Tumor size (cm)		2.5 ± 1.2	5.4 ± 3.5	.001
Type of resection				
1 Segment		97 (83.6%)	38(18.3%)	<.001*
2 Segment		19 (16.4%)	170(81.7%)	
Operation time (minutes)		156.3 ± 308.2	190.9 ± 79.2	.126
Blood loss (mL)		138.9 ± 336.0	1147.4 ± 1649.4	<.001*
Transfusion	No	108	102	<.001*
	Yes	8	106	
Blood transfused (mL)		47.4 ± 174.2	658.7 ± 1298.3	<.001*
Mortality	No	116	202	.092
	Yes	0	6	
Complication	No	109	145	<.001*
	Yes	7	63	
